# Histopathological and Biochemical Assessment of Neuroprotective Effects of Sodium Valproate and Lutein on the Pilocarpine Albino Rat Model of Epilepsy

**DOI:** 10.1155/2021/5549638

**Published:** 2021-06-03

**Authors:** Aziza Rashed Al-Rafiah, Khlood Mohammed Mehdar

**Affiliations:** ^1^Medical Laboratory Technology Department, Faculty of Applied Medical Sciences, King Abdul Aziz University, Saudi Arabia; ^2^Department of Anatomy, Faculty of Medicine, Najran University, Najran, Saudi Arabia

## Abstract

Epilepsy is one of the most frequent neurological disorders characterized by an enduring predisposition to generate epileptic seizures. Oxidative stress is believed to directly participate in the pathways of neurodegenerations leading to epilepsy. Approximately, one-third of the epileptic patients who suffer from seizures do not receive effective medical treatment. Sodium valproate (SVP) is a commonly used antiepileptic drug (AED); however, it has toxic effects. Lutein (L), a carotenoid, has potent antioxidant and anti-inflammatory properties. The aim of this study was to determine the neuroprotective effect of sodium valproate (SVP) and lutein (L) in a rat model of pilocarpine- (PLC-) induced epilepsy. To achieve this aim, fifty rats were randomly divided into five groups. Group I: control, group II: received PLC (400 mg/kg intraperitoneally), group III: received PLC + SVP (500 mg/kg orally), group IV: received PLC + SVP + L (100 mg/kg orally), and group V: received (PLC + L). Racine Scale (RC) and latency period to onset seizure were calculated. After eight weeks, the hippocampus rotarod performance and histological investigations were performed. Oxidative stress was investigated in hippocampal homogenates. Results revealed that SVP and L, given alone or in combination, reduced the RC significantly, a significant delay in latency to PLC-kindling onset, and improved rotarod performance of rats compared with the PLC group. Moreover, L was associated with a reduction of oxidative stress in hippocampal homogenate, a significant decrease in serum tumor necrosis factor-alpha (TNF-*α*) level, and inhibition of cerebral injury and displayed antiepileptic properties in the PLC-induced epileptic rat model. Data obtained from the current research elucidated the prominent neuroprotective, antioxidant, and anti-inflammatory activities of lutein in this model. In conclusion, lutein cotreatment with AEDs is likely to be a promising strategy to improve treatment efficacy in patients suffering from epilepsy.

## 1. Introduction

Epilepsy is one of the most common neurological disorders. Intermittent episodes of exaggerated, atypical cerebral activity give rise to the clinical features, ranging from debilitating fits, cerebral processing abnormalities, and mental health issues to the life-threatening clinical syndrome of status epilepticus (SE) [1]. The World Health Organization (WHO) figures indicate that epilepsy has a global incidence of 2.4 million and a prevalence of 50 million, affecting individuals regardless of age. Epilepsy is, therefore, a highly topical and relevant neurological cause of morbidity [2].

It is assumed that cerebral injury occurs following seizures, e.g., during SE, but this phenomenon's underlying pathophysiology remains obscure. Theories regarding factors that may exacerbate cerebrovascular damage include oxidative compromise, neuronal inflammation, and the development of atypical axonal connections. Such issues may precipitate a loss of higher cortical function [3].

To comprehend the pathophysiology and etiology of preictal and epileptic states and develop novel, efficacious, and low-risk therapeutic alternatives, it is essential to have a research tool to simulate the clinical situation. Pilocarpine (PLC) injection in rats or mice is a frequently used and long-standing experimental means to create SE and provoke ongoing epileptiform seizures in animals for research purposes [4].

A typical therapeutic agent for treating epilepsy is sodium valproate; its use is advocated to counter a large spectrum of seizure types. However, its efficacy may be compromised by its adverse reaction profile [5], which includes two particularly significant issues, i.e., risk of embryo malformation in pregnancy [6] and toxic effects on the liver [7]. There is a veritable pharmacy of antiepileptic drugs (AED) in clinical use, either as single agents or in combination. However, only two-thirds of patients respond to such treatment, leaving a significant cohort potentially immune to conventional therapy, despite trials of varying drug regimes. Additionally, an adverse side effect profile is an issue with several currently employed AEDs [8]. Cerebral damage that may be initiated by SE is not prevented by drug use [9]. Furthermore, some of the more traditional AED may break down to provide pharmaceutically active components, e.g., reactive oxygen species (ROS) that may exacerbate neuronal intracellular oxidative stress, contributing to the injurious process [10].

Lutein (L), a nonsynthetic carotenoid with antioxidant and inflammatory activity and has less toxic effects, has an improved risk: benefit ratio compared with more traditional antiepileptics such as SVP. L is a xanthophyll; vegetables such as carrots, spinach, and kale contain large amounts [11]. A specific subatomic configuration confers L with significant antioxidant properties [12]. L and its isomer, zeaxanthin, are the sole carotenoids with the ability to pass through the blood-retina barrier into the eye, where they are utilized for retinal macular pigment production protective antioxidant. Also, L can cross the blood-brain barrier in humans of any age where it is absorbed into cerebrovascular cells [13]. Together with its antioxidant and anti-inflammatory properties, this ability makes L an ideal candidate for study as a potential antiepeliptic agent.

The aims of this study are to assess the potential antiepileptic properties and neurological consequences of L in albino rats with PLC-induced epilepsy, to evaluate the effect of L on oxidative stress precipitated by evoked seizures, and to investigate any potential interactions or consequences when administered with SVP, a more conventional AED.

## 2. Materials and Methods

### 2.1. Experimental Animals

Fifty male albino rats, aged 7-9 weeks, mass 200-500 g, were supplied by The Animal House, King Fahad Medical Research Center, King Abdul-Aziz University, Jeddah, Saudi Arabia. Rodents were cared for in purpose-built transparent polycarbonate enclosures. Water was readily available; illumination source simulating night/day pattern was established. Ethical approval was obtained from the institution's Bioethics and Research Committee under the reference number (442-36-7263).

### 2.2. Drugs and Chemicals

Sodium valproate (SVP) (CAS number:1069-66-5), pilocarpine hydrochloride (PLC), and lutein (L) were procured from CaroteNature (CAS number No. 0133.1), (Zeaxanthin-free), (Lupsingen, Switzerland). Murine serum tumor necrosis factor-alpha (TNF-*α*) levels were assayed utilizing an enzyme-linked immunosorbent assay (ELISA) kit (Catalog Number: MBS825075). Additional ELISA assays used included kits to detect rat reduced glutathione (RGLU) (Catalog Number: MBS267424), rat malondialdehyde (MDA) (Catalog Number: MBS738685), and glutamate concentration (Glut) (Catalog Number: MBS269969). A reactive oxygen species (ROS) assay kit (Catalog Number: MBS2540517) was also acquired. MyBioSource, Inc. San Diego, USA, supplied all respective biochemical reagent packages.

### 2.3. Experimental Groups

Rats were acclimatized for seven days in their surroundings. Five groups of ten rats each were randomly as follows:

Group I (negative control): distilled water was orally administered daily via gavage. Intraperitoneal injection of 1 ml normal saline was injected three times per week.

Group II (positive control): intraperitoneal injection of PLC, 400 mg/kg, was given three times per week.

Group III (PLC/SVP): oral gavage was used to deliver 500 mg/kg SVP once daily. Intraperitoneal PLC, 400 mg/kg, was injected three times per week.

Group IV (PLC/SVP/L): 100 mg/kg L, dissolved in distilled water, and 500 mg/kg of SVP were fed via oral gavage daily. PLC, 400 mg/kg, was also administered via peritoneal injection three times per week.

Group V (PLC/L): 100 mg/kg L, dissolved in distilled water, was delivered once a day through oral gavage. Intraperitoneal PLC injections were given three times per week.

Previous studies were used to guide the dosage of PLC [13], SVP [14], and L [15].

### 2.4. PLC-Induced Kindling

400 mg/kg PLC dissolved in 1 ml of normal saline was injected via the intraperitoneum three times per week for no longer than eight weeks to evoke kindling. Following each injection, rats were then monitored individually in a clear, isolated box for 30 minutes. Any seizure was classified according to the Racine Score (RC) [16], i.e., 0-no response, 1-myoclonic jerks without rearing, 2-myoclonic jerks with rearing, 3-unilateral forelimb clonus, 4-rearing with bilateral forelimb clonus, and 5-generalized tonic-clonic seizure (GTCS). If the RC score reached stage 4 or 5 during three consecutive assessments, the rat was judged to have kindled. During the 8th week, latency to onset and duration of generalized seizures were timed [17].

### 2.5. Experimental Behavioral Parameters

The following behavioral parameters were measured:

PLC-induced kindling: this was derived from the time periods in days for stimulation to each level. The mean kindling score was also determined [18].

Motor function and grip strength: on a day when no PLC injection was scheduled, the rotarod performance test was used to assess the locomotor function and proprioception. This was conducted in a separate laboratory; rodents were given a minimum of an hour to adjust to test conditions. The assessment required the rats to cross a 7 cm diameter rod, rotating at a constant speed of 12 rpm, for at least 1 minute. Rats from the same enclosure were assigned individual tracks. Three attempts were made on the rod with a 5-minute rest period between each. Equipment was cleaned after each use. Following the final PLC dose, the test was conducted on each rat individually. Latency period to fall, i.e., the duration of each rat was able to sustain walking along the rod, was timed to a maximum of 3 minutes [19].

### 2.6. Histological and Immunohistochemical Studies for Light Microscopy

After the experiment, peripheral blood samples were taken; all rats were then sacrificed by cervical dislocation. A coronal slice acquired brain specimens for further testing. Samples were embedded in paraffin after being fixed in 10% buffered formalin, dried, and cleared. Serial sections, thickness five *μ*m, underwent staining with hematoxylin and eosin (×H&E) and toluidine blue to prepare light microscopy [20]. The samples were treated with an avidin-biotin peroxidize technique to detect astrocyte glial fibrillary acidic protein (GFAP) and then counterstained with hematoxylin. A reaction giving rise to a brown hue indicated the presence of GFAP [21].

### 2.7. Outcome Parameters

The following outcome parameters were assessed:

Serum tumor necrosis factor-alpha (TNF-*α*): prior to sacrifice, retroorbital blood samples were acquired. The sample was allowed to clot at room temperature by standing for 30 minutes. Serum was separated to permit the TNF-*α* assay by 15 minutes centrifugation at 3,500 rpm.

Hippocampal tissue homogenate sample preparation [16]: hippocampal tissue (100 mg) was acquired from each rats of each group and washed with 1× phosphate buffered saline (PBS). It was in homogenized in 1 ml of ice-cold 1× PBS and frozen overnight at -20°C. Two freeze-thaw cycles were conducted in order to fracture cell membranes. Centrifugation was then performed for 5 minutes at 5,000 rpm and temperature of 2-8°C; supernatant was extracted and calibrated immediately. Aliquot samples were stored at -20°C or -80°C. After further thawing and centrifuging, the following assays were run: reduced glutathione (GSH), lipid peroxidation (MDA), reactive oxygen species (ROS), and glutamate concentration (Glut) using the enzyme-linked immunosorbent assay (ELISA) kit. ELISA kit horseradish peroxidize conjugate was used to precipitate the enzymatic reaction. Spectrophotometry was employed to measure color intensity; this was inversely proportional to kit concentration. Standard curves were generated for each assay.

### 2.8. Statistical Analysis

IBM SPSS Statistics for Windows, version 21 (IBM Corp., Armonk/N.Y., USA) was used for statistical analysis. The mean and standard error (SE) was used to report descriptive data. Prism software version 5 was utilized to plot graphs. Pearson's chi-square test was used to evaluate intergroup differences. Statistical significance was defined as *p* value of less than 0.05.

## 3. Results

### 3.1. Effects of Tested Drugs on Seizure Score in the PLC-Kindling Rat Model

Compared with the normal controls, RC's sustained rise was noticed in groups receiving PLC (Table 1).

A significant reduction in RC (*p* = 0.01) was seen in the SVP/PLC animals compared with the positive controls over the eight weeks. RC was also diminished (*p* = 0.001) in those rats receiving L as a sole agent compared to both positive and negative control groups. The most significant reduction in the RC Score was seen when group IV, i.e., those rats receiving combination therapy of L and SVP, was assessed against groups I, II, and V (*p* = 0.0001).

A significant delay in the establishment of PLC kindling compared with the positive controls, 42.40 ± 1.84 days, was seen in the PLC/SVP and PLC/L groups, i.e., 50.30 ± 0.82 (*p* = 0.0001) and 47.10 ± 0.74 days, (*p* = 0.0001), respectively. The delay was even greater in rats receiving SVP and L, i.e., 58.60 ± 1.84 days. This was significant when compared against group II (*p* < 0.05) and group III animals (*p* = 0.0001).

**Figure 1:**
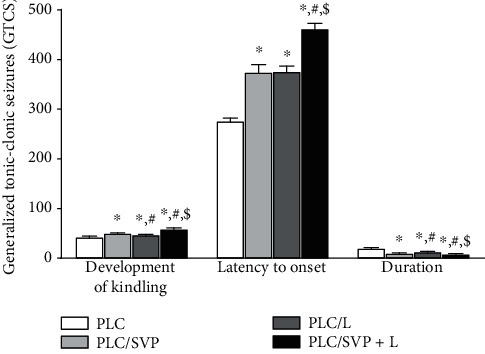
Latency to onset of GTCS and duration of GTCS in rats subjected to PLC-induced epilepsy. PLC: pilocarpine; SVP: sodium valproate; L: lutein. ^∗^Significance versus PLC; #significance versus PLC/SVP; $significance versus PLC/L.

The use of SVP was associated with a significant delay in latency to PLC-evoked GTCS onset; latency times in rats treated with PLC/SVP and the positive controls were 374.20 ± 15.55 and 276.00 ± 5.58 seconds, respectively (*p* = 0.0001) (Figure 1).

A similar finding was seen with the L administration: latency times for the PLC/L group were up to 375.90 ± 10.46 seconds (*p* = 0.0001). An even more significant effect was seen with SVP and L's combined therapy than groups II and III; in group V, the latency delay was up to 461.90 ± 10.55 seconds (*p* = 0.0001).

By the end of the experiment period, a shortening of seizure length was observed in rats treated with SVP, i.e., group III, 9.80 ± 0.92 seconds, compared with 19.70 ± 1.42 seconds in the positive controls (*p* = 0.0001). A similar effect was seen in group V where subjects were treated with L alone; seizure duration was 12.80 ± 0.92 seconds, again significantly reducing compared with group II. The decrease in seizure time seen was even more marked in group IV rats treated with both agents than the positive controls, i.e., 8.30 ± 0.82 seconds (*p* = 0.001).

### 3.2. Effects of Tested Drugs on Motor Performance of PLC-Kindling Animals: Rotarod Test

**Figure 2:**
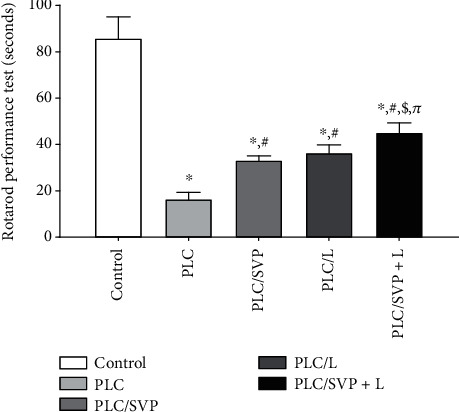
Rotarod performance test (latency time) (seconds). PLC: pilocarpine; SVP: sodium valproate; L: lutein. ^∗^Significance versus control, #significance versus PLC, $significance versus PLC/SVP, and *π*significance versus PLC/L.

Figure 2 illustrates the performance duration of the rotarod, which was diminished in PLC-kindling rats compared with the negative controls, i.e., 16.42 ± 2.90 versus 85.73 ± 9.33 seconds, respectively (*p* = 0.0001). Loss of motor activity and proprioception skills was noted in the former. SVP improved rats' locomotion skills compared with the positive control group with a rod duration achieved of 33.10 ± 1.94 seconds (*p* = 0.0001). An even more significant effect was seen in those rats receiving combination therapy, i.e., 36.42 ± 3.34 seconds, a significant increase in performance time when compared with both PLC controls (*p* = 0.0001) and those treated with SVP alone (*p* = 0.0001).

### 3.3. Histopathological Changes in the Model of PLC-Kindling: Effects of Various Treatments

**Figure 3:**
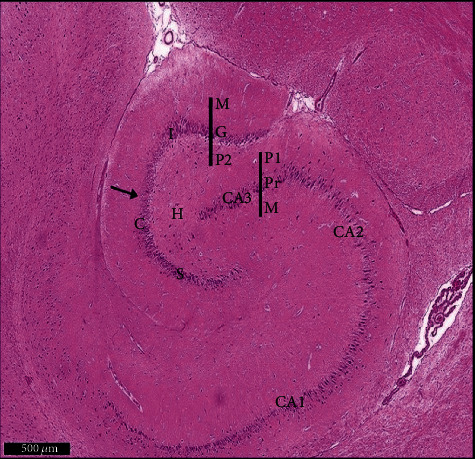
Representative histological appearance of hippocampus proper layers and dentate gyrus. The outer is layer polymorphic (P1), the middle pyramidal layer (Pr), and the inner molecular layer (M) in CA1, CA2, and CA3. The dentate gyrus (↑) shows crest (C), suprapyramidal (S), and infrapyramidal blades (I). Note that the area Cornu ammonis (CA3) surrounded by hilus (H). Polymorphic layer (P1) in DG. Scale bar: 500 *μ*m, negative control group I, H&E, ×5.

**Figure 4:**
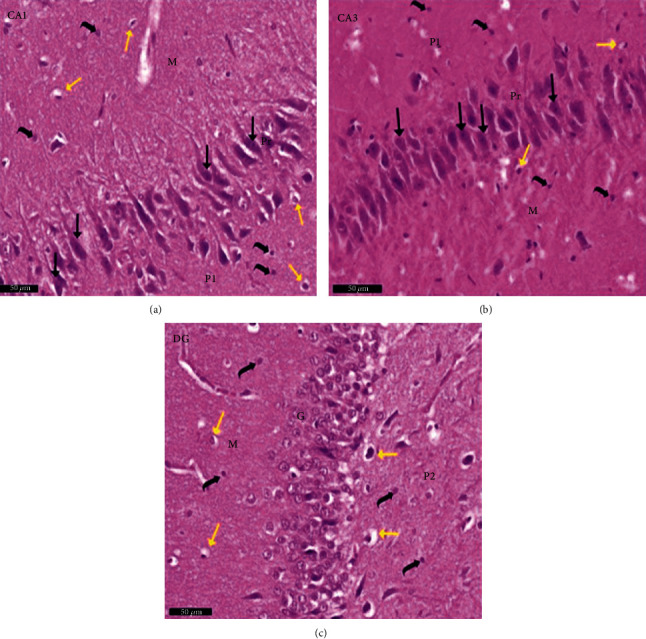
A photomicrograph of the hippocampus form albino rat group I (negative control group). Images show the (a) CA1 and (b) CA3 areas of hippocampus proper and (c) dentate gyrus. CA areas are formed of three layers: molecular layer (M), pyramidal (Pr), and polymorphic (P1) layers. Molecular layer is a relatively cell-sparse area. Pyramidal cells are arranged in the compact layer with little neuropil between the cells. They are seen with basophilic cytoplasm (↑) and vesicular nuclei. The granule cell layer (G) of the dentate gyrus formed mainly of uniformly sized closely packed granule cells with rounded cell bodies and vesicular nuclei. Notice glial cells (yellow arrow) and deeply stained interneurons (curved black arrow) in molecular and polymorphic layers (P1 and P2). Scale bar: 50 *μ*m, group I, H&E ×20.

**Figure 5:**
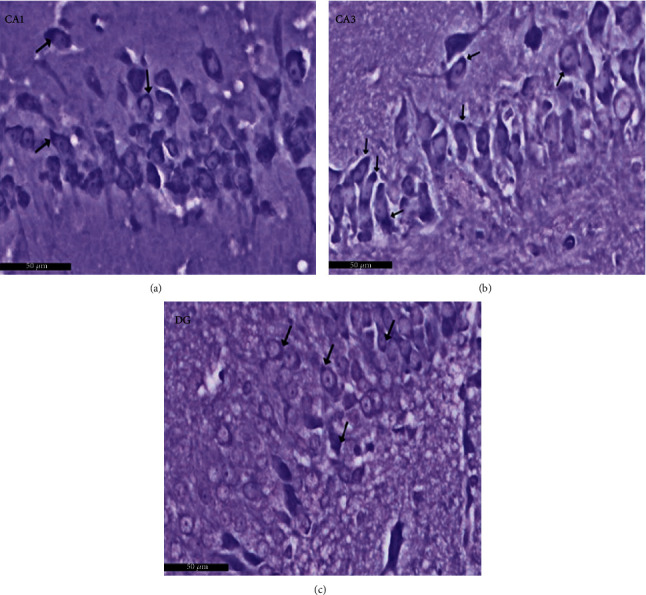
A photomicrograph of the CA1, CA2, and DG regions of hippocampus of albino rat of group I (negative control group). The images show the (a) CA1 and (b) CA3 areas of hippocampus proper and (c) dentate gyrus. The Nissl granules appear heavily studded in pyramidal and granule cell cytoplasm (↑). Scale bar: 50 *μ*m, group I, toluidine blue stain ×40.

**Figure 6:**
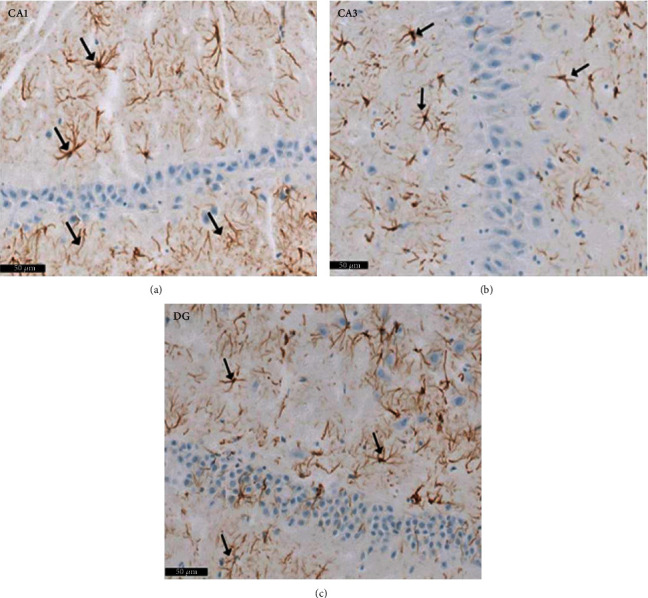
A photomicrograph of the hippocampus of albino rat from group I (negative control group). Images show positive GFAP brownish cytoplasmic reaction in astrocytes. Small cell bodies with short cytoplasmic processes of (a) CA1 and (b) CA3 areas of hippocampus proper and (c) dentate gyrus. (↑) is seen. Scale bar: 50 *μ*m, negative control group I, avidin–biotin technique GFAP, ×20.

Group I (negative controls). The three territories of the cornu ammonis (CA) that form part of the HP were identified on the H× & E stained slides, i.e., CA1, superiodistal and bordering with the dentate gyrus (DG); CA3, interproximal and lying adjacent to the subiculum; and CA2, situated between CA1 and CA3. Three strata of uniform histological appearance were identified throughout the HP (Figure 3). The most prominent layer consisted of pyramidal cells, characterized by central vesicular nuclei and basophilic cytoplasm (Figure 4). Surrounded by CA3, the DG was observed as a V-shaped territory, made up of inner suprapyramidal and outer infrapyramidal blades, connected by the DG crest (Figure 3). The polymorphic, granular cell and molecular layers of the DG were evident. Small-sized densely packed granule cells with rounded vesicular nuclei made up the granular layer (Figure 4). Nissl staining highlighted the large population of Nissl granules within the pyramidal cells (Figure 5). Astrocytes, i.e., GFAP-positive cells, were identified by a dark brown hue in response to staining with avidin-biotin; they were noted in CA1, CA3, and DG territories (Figure 6). Numerous short cytoplasmic processes on modestly sized cells were noted.

**Figure 7:**
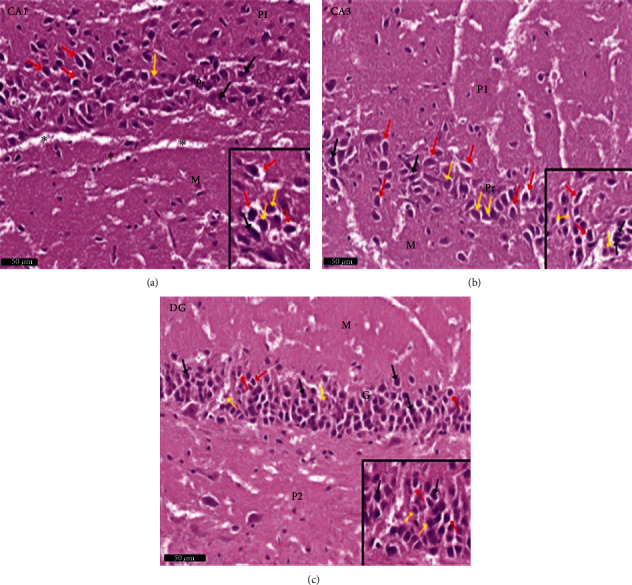
Photomicrographs of the hippocampus of albino rat group II (positive control group) show CA1, CA3, and DG. The image shows the (a) CA1 and (b) CA3 areas of hippocampus proper and (c) dentate gyrus. CA areas are formed of three layers: molecular layer (M), pyramidal (P), and polymorphic (P1) layers. In CA2 and CA3 areas, shrunken pyramidal cells with shrunken deeply stained pyknotic nuclei and deeply stained basophilic cytoplasm (↑) with perineuronal spaces within the pyramidal layer (red arrow) are seen, in CA1 and CA3 area pyramidal cells with karyolitic pale stained nuclei (yellow arrow). Dentate gyrus is formed of the molecular (M) layer, granule cell layer (G), and polymorphic layers (P2). An apparent decrease thickness of the granule cell layer is noticed compared to the control group. Shrunken granule cells with shrunken deeply stained pyknotic nuclei and deeply stained basophilic cytoplasm (↑) are seen. Halos are noticed around degenerated granule cells (red arrow). Some granule cells are seen with karyolitic nuclei (yellow arrow). Notice the pale vacuolated areas (^∗^) in the molecular layer (M). Inset: higher magnification of the pyramidal cells and granular cells. Scale bar: 50 *μ*m, positive control group II, H&E ×20 and inset ×40.

**Figure 8:**
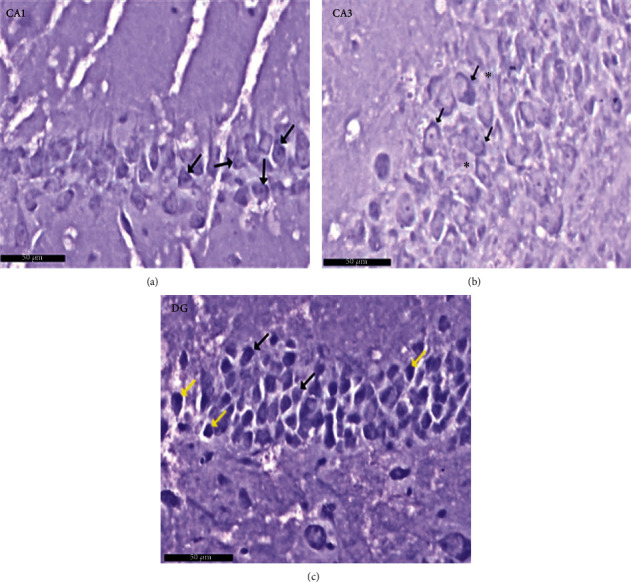
Photomicrographs of the hippocampus of albino rat group II (positive control group). The image shows the (a) CA1 and (b) CA3 areas of hippocampus proper and (c) dentate gyrus and shows CA1, CA3, and DG. An apparent decrease of the Nissl granules is seen in pyramidal and granule cells (↑) and ill-defined margins in CA1 and CA3 areas as compared to group I (negative control). In the CA3 area, wide inter cellular spaces are seen between pyramidal cells (^∗^). In DG, cells with deep basophilic cytoplasm (yellow arrow) are noticed. Scale bar: 50 *μ*m, positive control group II, toluidine blue stain ×40.

**Figure 9:**
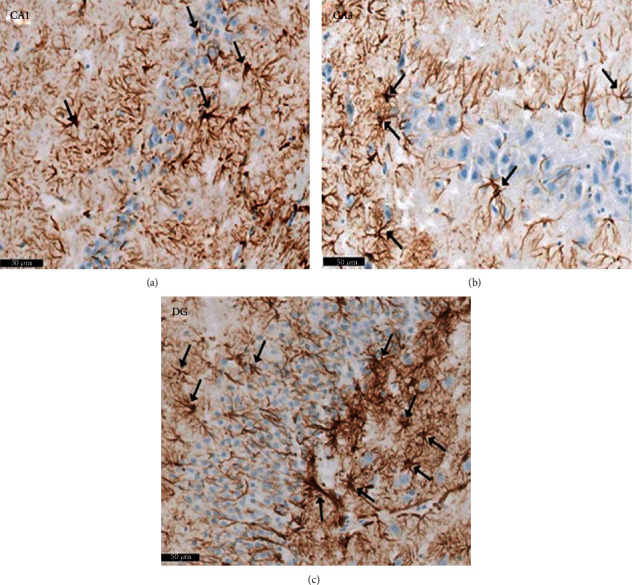
Photomicrographs of the hippocampus of albino rat group II (positive control group) showing GFAP positive astrocytes (↑) in polymorphous and molecular layer. An apparent increase number of astrocytes are noticed in (a) CA1, (b) CA3, and (c) DG areas. Glial fibers appear twisted, thickened, and intensely stained compared to the control group. Scale bar: 50 *μ*m, positive control group II, GFAP immunostaining ×20.

Group II (positive controls): Figure 7 depicts the pyramidal layer neuronal atrophy, stained dark by H× & E, and perineuronal spaces identified in CA1 and CA3 areas. Compared with the negative control group, karyolitic pale stained nuclei were noted in pyramidal cells from the same anatomical territories. The granule cell layer of the DG was reduced in thickness; attenuated pyknotic nuclei and basophilic cytoplasm stained heavily. A striking halo appearance was observed surrounding degenerated granule cells, some of which contained karyolitic nuclei. Pyramidal cell axonal edema and degeneration were intimated by the evidence of pale vacuolated regions in the molecular area. Nissl granule presence was diminished in CA1, CA3, and DG granule cells (Figure 8) compared to equivalent Nissl-stained slides from the negative control rats. Astrocytes identified by avidin-biotin staining exhibited more extensive cytoplasmic processes (Figure 9). Compared to group I, twisting and thickening of the glial fibers were observed.

**Figure 10:**
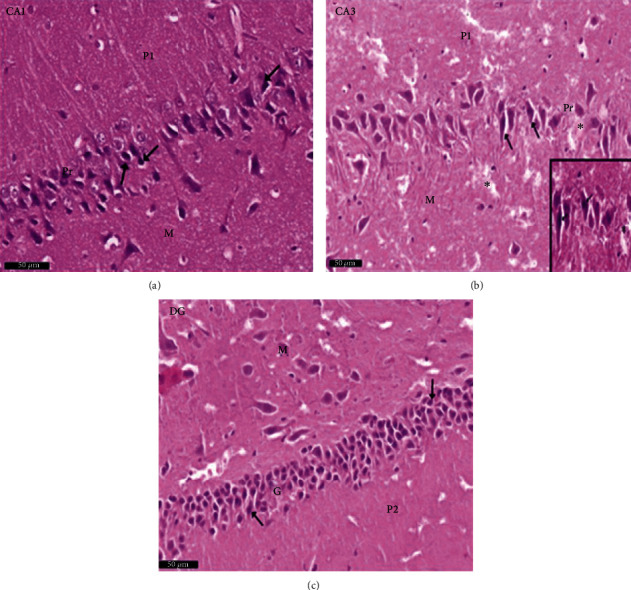
Photomicrographs of the hippocampus of albino rat group III (PLC/SVP) show (a) CA1, (b) CA3, and (c) DG. CA areas are formed of three layers: molecular layer (M) and pyramidal (P) and polymorphic (P1) layers, in CA2 and CA3 areas, occasional shrunken darkly stained pyramidal cells (↑). Dentate gyrus is formed of molecular (M) layer, granule cell layer (G), and polymorphic layers (P2). Occasional shrunken granule cells with shrunken deeply stained pyknotic nuclei (↑) are seen. Inset: higher magnification of the pyramidal cells in the CA3 area. Scale bar: 50 *μ*m, PLC/SVP group III, H&E ×20 and inset ×40.

**Figure 11:**
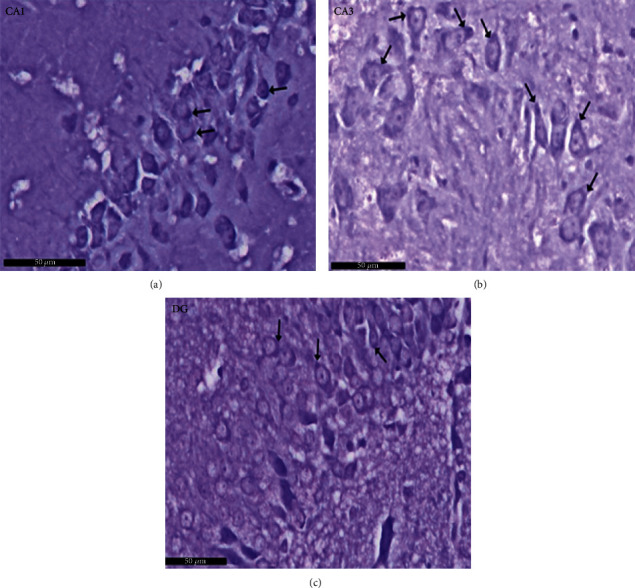
Photomicrographs of the hippocampus of albino rat from group III (PLC/SVP) reveal (a) CA1, (b) CA3, and (c) DG. An apparent increase of the Nissl granules is seen in pyramidal and granule cells (↑) as compared to group II (positive control group). Scale bar: 50 *μ*m, group III (PLC/SVP), toluidine blue stain ×40.

**Figure 12:**
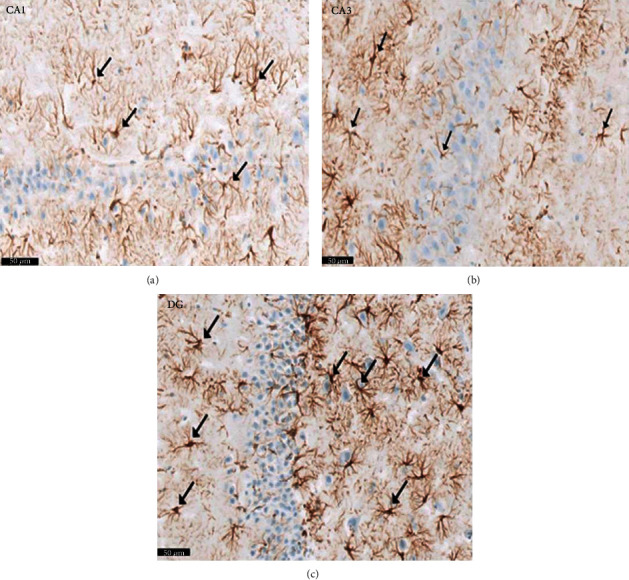
Photomicrograph of the CA1, CA3, and DG regions of the hippocampus of a rat group III (PLC/SVP). The images show (a) CA1, (b) CA3, and (c) DG. They reveal apparent decrease of the positive GFAP brownish reaction. Notice that astrocytes present shorter processes (↑) and decrease in the intensity of GFAP immunostaining compared to that of group II (Epileptic model group). Scale 50 *μ*m, group III (PLC/SVP), GFAP immunostaining ×20.

Group III (PLC/SVP): the anatomical areas CA1, CA3, and DG were identified on H× & E microscopy (Figure 10). Pyramidal cells demonstrating attenuation and that stained darkly were rarely seen in CA2 and CA3. In contrast to the positive controls, the granule cell layer was hypertrophied. Granule cells that were reduced in size with vestigial heavily staining pyknotic nuclei were few and far between.  Figure 11 illustrates the increase in Nissl staining identified in both pyramidal and granule cells compared with that seen in group II rats. A reduction in astrocytes, detected with immunostaining for GFAP, was seen compared with both control groups. Reduced size and diminished length of cytoplasmic processes were noted in those astrocytes identified (Figure 12).

**Figure 13:**
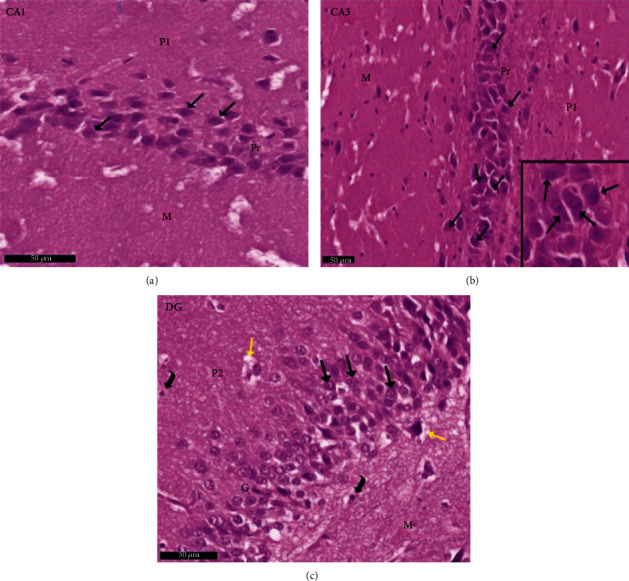
A photomicrograph of the hippocampus of albino rats from group IV (PLC/SVP/L) shows (a) CA1, (b) CA3, and (c) DG areas. They appeared nearly as the negative control group. Notice glial cells (yellow arrow) and deeply stained interneurons (curved black arrow) in the molecular (M) and polymorphic layer (P2). Inset: higher magnification of the pyramidal cells in the CA3 area. Scale bar: 50 *μ*m, group IV (PLC/SVP/L), H&E ×20.

**Figure 14:**
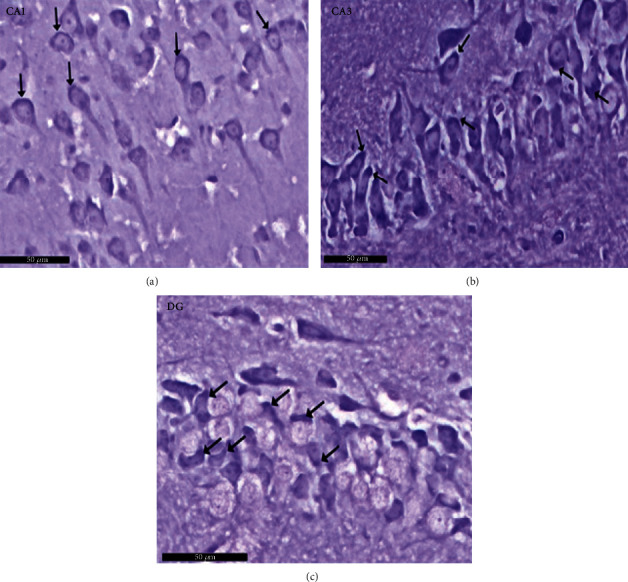
Photomicrographs of the hippocampus of albino rat from group IV (PLC/SVP/L) show (a) CA1, (b) CA3, and (c) DG. An apparent increase of the Nissl granules is seen in pyramidal and granule cells (↑) as compared to group II (positive control group). Scale bar: 50 *μ*m, group IV (PLC/SVP/L), toluidine blue stain ×40.

**Figure 15:**
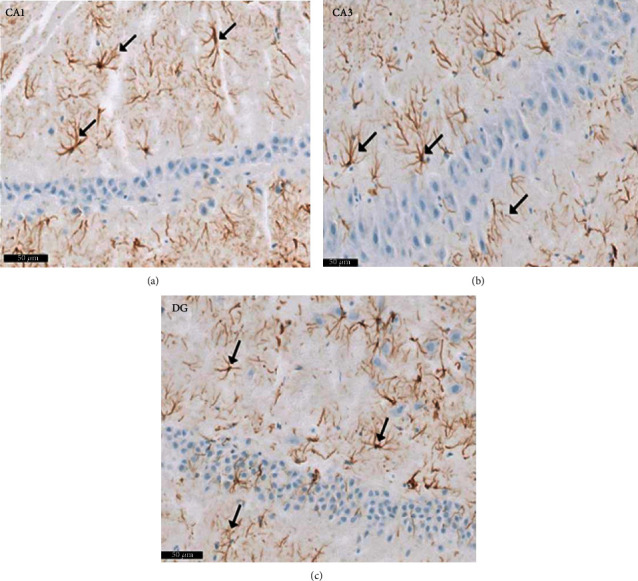
A photomicrograph of a rat group IV (PLC/SVP/L). The images show (a) CA1, (b) CA3, and (c) DG. Reveal apparent decrease of the GFAP positive brownish reaction. Notice that astrocytes present shorter processes (↑) as well as decrease in the intensity of GFAP compared to that of group II (Epileptic model group) and group III (PLC/SVP). Scale 50 *μ*m, group IV (PLC/SVP/L), GFAP immunostaining ×20.

Group IV (PLC/SVP/L): in group IV samples, H&E staining revealed nearly regular microscopic appearances.  Figure 13 illustrates the presence of the typical three strata of CA1 and CA3 areas. Central vesicular nuclei and basophilic cytoplasm were seen within the pyramidal cells from these territories; the DG granule cell layer also retained its structure (Figure 13), although when compared with the negative control group, its thickness was augmented. In the molecular and polymorphic levels, the presence of glial cells and heavily staining interneurons was identified. Pyramidal and granule cells showed a higher concentration of Nissl granules, as seen on Nissl staining than the equivalent slides in group II rats (Figure 14). Compared with the positive controls and those treated with SVP alone (group III), an increase in Nissl granule density and a reduction in the astrocyte population were noted (Figure 15).

**Figure 16:**
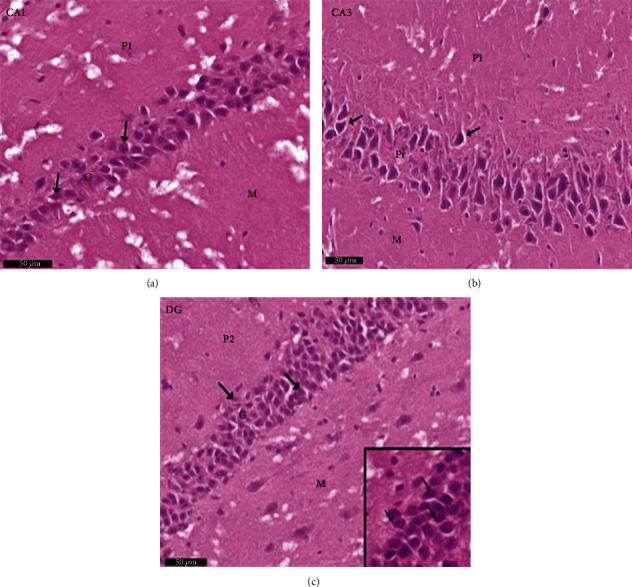
Photomicrographs of the hippocampus of albino rat group V (PLC/L) reveal (a) CA1, (b) CA3, and (c) DG. CA areas are formed of three layers. Most of pyramidal cells are seen with basophilic cytoplasm (↑) and vesicular nuclei. Few dark stained neurons with perineural space are seen in the pyramidal layer of CA3 (↑). Dentate gyrus is formed of the molecular (M) layer, granule cell layer (G), and polymorphic layers (P2). Most of granular cells are seen with basophilic cytoplasm (↑) and vesicular nuclei. Inset: higher magnification of the granular cells. Scale bar: 50 *μ*m, group V (PLC/L), H&E ×20 and inset ×40.

**Figure 17:**
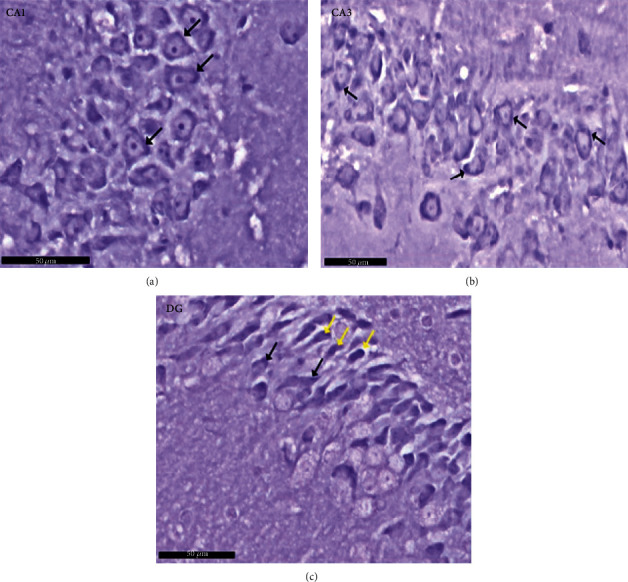
Photomicrographs of the hippocampus of albino rat from group V (PLC/L) show (a) CA1, (b) CA3, and (c) DG. An apparent increase of the Nissl granules is seen in pyramidal and granule cells (↑) as compared to group II (positive control group). In DG, cells with deep basophilic cytoplasm (yellow arrow) are noticed. Scale bar: 50 *μ*m, group V (PLC/L), toluidine blue stain ×40.

**Figure 18:**
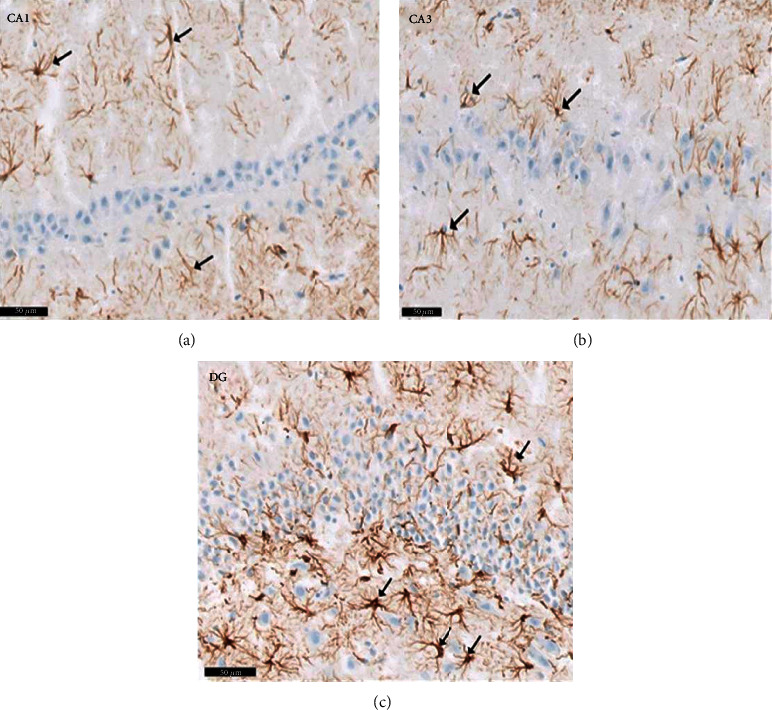
A photomicrograph of a rat group V (PLC/L). The images show (a) CA1, (b) CA3, and (c) DG, showing apparent decrease of the GFAP positive brownish reaction. Notice that astrocytes present shorter processes (↑) as well as decrease in the intensity of GFAP compared to that of group II (Epileptic model group) and group III (PLC/SPA), in the DG showing an apparent increase of the positive brownish reaction for GFAP as compared to group I (control negative). Scale 50 *μ*m, group V (PLC/L), GFAP immunostaining ×20.

Group V (PLC/L): histological integrity of the CA1 and CA3 territories was noted on H× & E stained samples (Figure 16), preserving the central vesicular nuclei and basophilic cytoplasm in the pyramidal cells. Compared with the positive control group and the rats treated with SVP as a single agent, only a small number of atrophic and darkly staining neurons and perineuronal spaces were seen. Figure 16 also illustrates the appearance of the granule cells. These were consistent and noted to be dense in population; cell bodies were rounded, and vesicular nuclei were observed. A larger presence of Nissl granules in both pyramidal and granule cells was detected on Nissl staining compared to that seen in groups II and III (Figure 17). Additionally, GFAP staining revealed reduced size astrocytes characterized by shorter cytoplasmic processes. In the DG, astrocytes stained for GFAP showed a more marked cytoplasmic brown hue than the negative controls (Figure 18).

### 3.4. Effect of Treatments on TNF-Alpha

**Figure 19:**
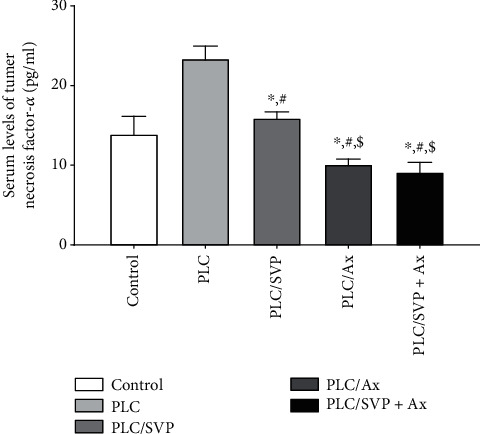
Serum levels of tumor necrosis factor-*α* (TNF-*α*). PLC: pilocarpine; SVP: sodium valporate; L: lutein. ^∗^Significance versus control, #significance versus PLC, and $significance versus PLC/SVP.

TNF-*α* concentration was significantly elevated in the positive controls receiving PLC (group II): 23.36 ± 1.5 pg/ml, compared with the negative controls (group I): 13.84 ± 2.3 pg/ml (*p* = 0.0001). Levels of TNF-*α* were decreased by delivery of SVP alone: 15.88 ± 0.81 (*p* = 0.003) and L alone: 10.08 ± 0.69 pg/ml (*p* = 0.0001), and in group IV, both agents were administered: 9.11 ± 1.22 pg/ml (*p* = 0.0001), when compared with the PLC control group (Figure 19).

### 3.5. Effect of Various Treatments on Oxidative Stress and Glutamate Parameters in the Hippocampus Tissue Homogenate in PLC-Kindling in Rats

**Figure 20:**
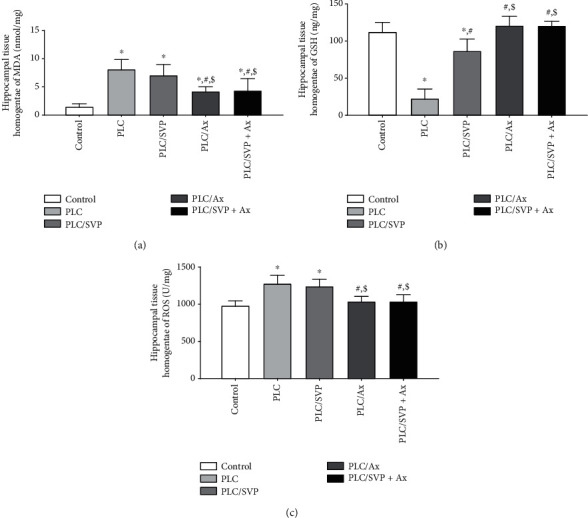
Oxidative stress markers in hippocampal tissues. PLC: pilocarpine; SVP: sodium valporate; L: lutein. (a) MDA: malondialdehyde; (b) GSH: reduced glutathione; (c) ROS: reactive oxygen species. ^∗^Significance versus control, #significance versus PLC, and $significance versus PLC/SVP.

A significantly increased MDA concentration was seen in the positive control group compared with the negative control group, i.e., 8.09 ± 1.76 and 1.44 ± 0.56 nmol/mg, respectively (*p* = 0.0001). MDA titers were reduced in group III: 7.04 ± 1.92 nmol/mg and V: 4.17 ± 0.88 nmol/mg, respectively, compared with group II (Figure 20(a)). Compared with the positive control group and the rats treated with SVP alone, a significantly lower MDA level was detected in the rats receiving both SVP and L: 4.33 ± 2.14 nmol/mg (*p* = 0.0001).

Figure 20(b) illustrates the significant reduction in the antioxidant GSH seen in group II: 22.64 ± 12.91 ng/mg (*p* = 0.0001) and group III: 86.79 ± 15.77 ng/mg (*p* = 0.0001) compared with the negative control group: 112.21 ± 12.77. GSH levels measured in rats treated with L alone: 120.80 ± 12.30 ng/mg were equivalent to those receiving combination therapy: 120.47 ± 6.04 ng/mg. The rats receiving PLC/SVP/L: 120.47 ± 6.04 ng/mg demonstrated a significantly higher GSH concentration than those receiving SVP alone (*p* = 0.0001).

ROS concentration was significantly elevated (*p* = 0.0001), in hippocampal homogenate from group II: 1278.32 ± 112.83 U/mg and group III: 1242.70 ± 93.36 U/mg, compared with group I: 978.97 ± 66.22 U/mg (Figure 20(c)). This increase in the ROS level was not significant (*p* = 0.133) in comparison to that detected in rats treated with L: 1038.40 ± 65.15 U/mg or SVP and L combination: 1038.43 ± 87.63 U/mg. A decrease in ROS was noted in group V as opposed to group IV, but this did not reach statistical significance (*p* = 0.999).

**Figure 21:**
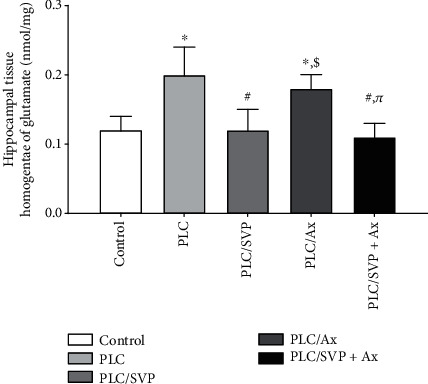
Glutamate concentrations in hippocampal tissues. PLC: pilocarpine; SVP: sodium valporate; L: lutein. ^∗^Significance versus control, #significance versus PLC, $significance versus PLC/SVP, and *π*significance versus PLC/L.

In comparison to normal controls, Glut was significantly elevated (*p* = 0.0001) in group II rats: 0.18 ± 0.02 and those treated with L: 0.20 ± 0.04 (Figure 21). When evaluated against group II animals, a notable finding was a significant reduction (*p* = 0.0001) in Glut in group III rats treated with SVP: 0.12 ± 0.03 and group IV rats undergoing combination treatment: 0.11 ± 0.02.

## 4. Discussion

This research evaluated L's potential antiepileptic and neuroprotective properties, evaluating its use as a single agent and in conjunction with SVP in a PLC-induced murine clinical model. In the subject groups, i.e., negative and positive (PLC) controls and those prescribed L, the RC and latency period to commence GTCS were documented. A notable finding was that rats receiving the combination of L and SVP demonstrated both lower RC and latency times than subjects in groups I, II, and V. This significant reduction in RC and more extended latency period to GCTS were not seen in any of the other groups. A conclusion that can, therefore, be drawn is that L has antiepileptic characteristics [22]. This finding is in line with demonstrating antiepileptic properties in crocin, a carotenoid indigenous to croci and gardenia blossom. Wang et al. [23] documented that 200 mg/kg of Crocin could be used to terminate epileptic seizures in fully kindled rats. The rate of loss of higher cerebral function in individuals with Alzheimer's dementia has been retarded using several dietary supplements, e.g., L and beta-carotene [24]. This clinical observation is consistent with the findings relating to the use of these agents in epilepsy.

Many researchers have used the rotarod test to evaluate murine locomotor and grip parameters [25]. It is a useful indicator of the disturbed locomotor ability and cognitive decline seen in epileptic subjects compared with healthy controls; typically, the former group shows lower test performance. The results obtained in this study are in keeping with locomotor impairment and behavioral issues precipitated by PLC-induced kindling [26]. The fact that both seizure grade and motor deficits improved in response to both L and SVP administration is a promising and encouraging finding, indicating that L can prove to be a higher AED than more typical agents. SVP, for example, moderates the ignition rate and favorably impacts grip strength loss associated with motor deficits [27].

In this research, biochemical assay results were correlated with the behavioral differences in the rodents. Hippocampal Glut was augmented in all PLC-kindling groups, but not in the negative control animals. Compared with group II, Glut was significantly reduced in rats receiving therapeutic agents, i.e., groups III and IV. Hippocampal injury has been postulated as a possible association between fits and memory loss; the above results align with this theory [28]. Release of excitatory neurotransmitters, e.g., glutamate, aspartate, and calcium ions, has also been cited in the literature as mediators of cerebral injury and “dark” neuron formation [28].

Li et al. [29] also reported a diminution in Glut and aspartate levels in a pentylenetetrazol- (PTZ-) induced kindling murine model. In a further study, L administration was found to prevent DNA breakdown in cerebrovascular injury; this was associated with reduced Glut. Reduced apoptosis was seen on terminal deoxynucleotidyl transferase dUTP nick end labeling [30].

The Glut is proposed as the main culprit for the induction of oxidative stress [10]. It acts as an excitatory neurotransmitter within the brain tissue, and specifically, when present in increased amounts, it may give rise to excitotoxicity. These properties may explain the findings in group II as opposed to those in PLC/SVP and PLC/SVP/L subjects, i.e., the elevated hippocampal levels of ROS and MDA and diminished GSH. Reeta et al. [31] noted equivalent findings; raised malonaldehyde concentration and reduced glutathione levels were measured in PLC-induced epilepsy in rats. Numerous further studies support that support these findings [8, 32]. Again in rat models of kindling induced by PLC and PTZ, an assay of hippocampal homogenate revealed diminished malonaldehyde and increased glutathione concentrations.

L's actions as a robust antioxidant in other pathological states are reported in the current literature [33, 34]. Long-term amygdala kindling in Sprague-Dawley mice were associated with catastrophic nerve cell degeneration in the CA3 territory [35]. Reactive oxygen specious and malonaldehyde levels were elevated, changes that were reversed by L administration. The latter also precipitated raised levels of glutathione. Neuronal damage was also significantly reduced by L. The authors concluded that L's neuroprotective effects in the epileptic hippocampus were the result of the following mechanisms: reduction in oxidative injury, peroxidation of lipids, and mitochondrial apoptotic pathway inhibition. A similar diminution of oxidative damage was noted in rats where epilepsy was evoked with kainic acid [15].

Limited cerebral territories can manufacture new nerve cells; however, the hippocampal DG is one of them [36]. The role of the DG in the onset of TLE is well recognized. The DG is a gatekeeper to monitor excitatory input into the hippocampus [37]. This rodent study draws attention to the histopathological alterations in the CA1 and CA3 territories and the DG when kindling is evoked. Besides, this is the first study to use a murine kindling model to evaluate the actions of L either administered singly or in conjunction with SVP, to compare these groups with the sole prescription of SVP and to conduct additional histological scrutiny of the HP and DG. Since the DG is a vital anatomical structure for data transfer to the HP, this cerebral territory is thought to be essential for normal higher cerebral function and the control of onset and incidence of epilepsy [38].

In this study, the positive control group displayed evidence of CA territory neurodegeneration, i.e., presence of pyramidal cells and granular cells within the DG, findings consistent with prior research. Additionally, the granular cell layer appeared thinner than in the control subjects. Cytopathological disturbances have been observed by Deng's group [39]. In a PLC-induced kindling murine model, they observed that the hippocampal pyramidal neurons were attenuated and displayed dark cytoplasm and pyknotic nuclei. According to Saha et al., 2014 [40], ROS may play an essential role in cell loss. Some workers have demonstrated DG neurogenesis following PTZ-kindling [39, 41, 42]. However, PTZ-kindling has also been associated with DG granular stratum attenuation and cell loss [34].

In the positive control group, the DG granule cells and CA1 and CA3 areas reduced the Nissl granule component.

In this research, compared to the normal controls, the examination of the CA1, CA3, and DG territories from the positive control group revealed high numbers of GFAP-positive astrocytes. Comparable discoveries have been made in corresponding anatomical areas in Wistar mice and male Sprague-Dawley rats [43–45]. Interestingly, this astrocyte increase was associated with the diminished population of normal nerve cells in these territories in rodents receiving six PTZ injections [46]. Heat shock protein- (HSP-) 27 is liberated from astrocytes; a rise in its concentration is associated with an increasing abundance of astrocyte cells. Thus, HSP-27 may be a useful biomarker for the detection of astrocytic oxidative stress [47].

Additionally, it can be used in PTZ-kindled rodents [48] and in TLE [49]. Kindled mice demonstrate significant GFAP-stain positive microscopy in the DG molecular layer [50]. A significant reduction in TNF-*α* was detected in group II positive controls compared with group I, which is consistent with previous research [51]. The cytokine, TNF-*α*, has long been recognized as stemming from microglia and astrocytes [52]. Therefore, it is postulated that this may be the underlying mechanism for the augmented astrocytic GFAP immunohistochemical expression seen in the studied brain samples of group II subjects. In the PLC-kindling rats, SVP appeared to reduce the severity of histopathological alterations; Nissl granule augmentation and a lower number of GFAP-positive astrocytes were identified. A comparable attenuation in histopathological change following valproic acid delivery was described by Sedky et al. [53] and also by Mannaa et al., who, in a PLC-induced epileptic model, additionally described an associated reduction in antioxidant levels [29].

A positive effect on histopathological findings caused by PLC-induced kindling was demonstrated in group IV, i.e., those treated with L and SVP, and group V, where L was given alone. Essentially, regular pyramidal cell appearances were present on H× and E stained microscopy of CA1 and CA3 territories; the DG granular cell layer showed no attenuation, even displaying augmented width, and again, the microscopic assessment was within normal limits, consistent with previously published findings [8, 31]. These workers concluded that in PTZ- and PLC-induced kindling, L has a neuroprotective action. L also attenuated epileptic activity in conjunction with the CA3 area reduced neuronal apoptosis [54]. This is facilitated by L's ability to cross the blood-brain barrier, thus enhancing its neuroprotective efficacy [55]. Therefore, L's actions may include antioxidative, anti-inflammatory, and antiapoptotic effects. Enhanced neurogenesis, somatic hypertrophy, or reactive astrogliosis may contribute to the DG granule layer augmentation demonstrated in this work. Varying studies have supported these mechanisms [8, 56].

Enhancement of neurogenesis and neuronal plasticity as potential effects of L has been proposed [57]. Neural progenitor cells (NPCs) may lead to novel neuron and circuit formation; thus, degenerating hippocampal cells are replaced. Stem cell activity is also present in the DG, where they are associated with preserving higher cerebral function caused by the facilitation of hippocampal neuronal regeneration. In in vitro experiments, NPC proliferation is stimulated by L [56]. L has also shown protective properties towards NPCs, in that it can inhibit apoptotic pathways initiated by exposure to oxidative stress [58]. The rise in Nissl granules identified in the DG granule cells and pyramidal cells from CA1 and CA3 territories seen in group IV (PLC/SVP/L) compared with group III (PLC/SVP) may be attributable to these L properties in the research presented. Wu et al. [69] documented similar results. They noted the augmented gene expression for both GFAP and brain-derived neurotrophic factor (BDNF) as a consequence of neurogenesis that occurred in response to prior therapy with L. BDNF is an essential component for synaptic nerve transmission in addition to neuronal regeneration [59]. GFAP has been identified as playing a prominent role in regeneration following cerebrovascular insult, cell communication, and the blood-brain barrier's efficiency [60].

The rise in the DG population of GFAP-positive cells in group V (PCL/L) is a notable finding. The process of glial scarring is often associated with an abundant astrocyte population gathered around an injury area [61], potentially inhibiting more widespread issues by isolating the affected territory. This phenomenon could therefore be responsible for the group V findings, observations that are reflected in studies that have reported diminution of cytokines, e.g., IL-1b and TNF-*α* following L delivery [62–64]. In a lithium-PLC model of SE, inflammatory cytokines titers were also attenuated following L administration [31]. The nuclear factor, *κ*B (NF *κ*B), is a proinflammatory transcription factor that mediates some of the actions of TNF-*α*. Cerebral exposure to stress causes NF-*κ*B activation, which in turn leads to upregulation of the expression of nitric oxide synthetase and the consequent production of nitric oxide, a potent cause of neurodegenerative injury [65]. Accumulation of intracellular ROS evoked by transforming growth factor-beta 1 (TGF-*β*1) can be inhibited by L. Potential inhibition of NF-*κ*B levels by L is likely to attenuate TGF-*β*1 activity [66].

## 5. Conclusions

The data from this research demonstrate that the antiepileptic properties of L act specifically on the hippocampus. Performance of tasks dependent on hippocampal integrity is improved by L administration; thus, L's therapeutic potential is related to its anti-oxidant, anti-inflammatory, and anti-apoptotic effects. These results add further evidence to the theory that L offers both neuroprotective and antiepileptic modes of action when prescribed singly or in conjunction with SVP in the PLC-induced kindling rodent model.

## Figures and Tables

**Table 1:** Racine score in rats subjected to PLC- induced epilepsy.

Racine Score	PLC	PLC/SVP	PLC/L	PLC/SVP + L
1^st^ week	1.83 ± 0.29	1.73 ± 0.09^1^P	1.73 ± 0.08 ^1^P, ^2^P	1.57 ± 0.20 ^1^P, ^2^P,^3^P
2^nd^ week	2.16 ± 0.21	1.77 ± 0.14^1^P	1.93 ± 0.08^1^P, ^2^P	1.67 ± 0.20^1^P, ^2^P,^3^P
3^rd^ week	2.54 ± 0.09	2.17 ± 0.14^1^P	2.15 ± 0.05^1^P, ^2^P	1.98 ± 0.12^1^P, ^2^P,^3^P
4^th^ week	2.77 ± 0.09	2.39 ± 0.02^1^P	2.33 ± 0.08^1^P, ^2^P	2.10 ± 0.05^1^P, ^2^P,^3^P
5^th^ week	3.47 ± 0.05	2.72 ± 0.17^1^P	3.05 ± 0.26^1^P, ^2^P	2.13 ± 0.03^1^P, ^2^P,^3^P
6^th^ week	4.11 ± 0.12	3.15 ± 0.38^1^P	3.17 ± 0.35^1^P, ^2^P	2.95 ± 0.32^1^P, ^2^P, ^3^P
7^th^ week	4.63 ± 0.08	3.40 ± 0.21^1^P	3.80 ± 0.32^1^P, ^2^P	3.12 ± 0.33^1^P, ^2^P,^3^P
8^th^ week	4.87 ± 0.05	4.06 ± 0.08^1^P	4.33 ± 0.18^1^P, ^2^P	3.87 ± 0.41^1^P, ^2^P, ^3^P

PLC: pilocarpine; SVP: valproic acid; L: lutein. ^1^PSignificance versus PLC, ^2^Psignificance versus PLC/SVP, and ^3^Psignificance versus PLC/L.

## Data Availability

The data available are in the manuscript.
